# Biochemical consequences of two clinically relevant ND-gene mutations in *Escherichia coli* respiratory complex I

**DOI:** 10.1038/s41598-021-91631-3

**Published:** 2021-06-16

**Authors:** Franziska Nuber, Johannes Schimpf, Jean-Paul di Rago, Déborah Tribouillard-Tanvier, Vincent Procaccio, Marie-Laure Martin-Negrier, Aurélien Trimouille, Olivier Biner, Christoph von Ballmoos, Thorsten Friedrich

**Affiliations:** 1 Institut für Biochemie, Albert-Ludwigs-Universität Freiburg, Freiburg, Germany; 2 IBGC, CNRS UMR5095, Bordeaux University, Bordeaux, France; 3 MitoLab, UMR CNRS 6015-INSERM U1083, Université d’Angers, Angers, France; 4 CHU de Bordeaux, 33000 Bordeaux, France; 5 Institut des Maladies Neurodégénératives, Univ. Bordeaux, 33000 Bordeaux, France; 6 CNRS, Institut des Maladies Neurodégénératives, 33000 Bordeaux, France; 7 Univ. Bordeaux, lNSERM U1211, 33000 Bordeaux, France; 8 Department of Chemistry and Biochemistry, University of Bern, Bern, Switzerland; 9 Present Address: MRC Mitochondrial Biology Unit, University of Cambridge, Cambridge, UK

**Keywords:** Multienzyme complexes, Oxidoreductases, Membrane proteins

## Abstract

NADH:ubiquinone oxidoreductase (respiratory complex I) plays a major role in energy metabolism by coupling electron transfer from NADH to quinone with proton translocation across the membrane. Complex I deficiencies were found to be the most common source of human mitochondrial dysfunction that manifest in a wide variety of neurodegenerative diseases. Seven subunits of human complex I are encoded by mitochondrial DNA (mtDNA) that carry an unexpectedly large number of mutations discovered in mitochondria from patients’ tissues. However, whether or how these genetic aberrations affect complex I at a molecular level is unknown. Here, we used *Escherichia coli* as a model system to biochemically characterize two mutations that were found in mtDNA of patients. The V253A^MT-ND5^ mutation completely disturbed the assembly of complex I, while the mutation D199G^MT-ND1^ led to the assembly of a stable complex capable to catalyze redox-driven proton translocation. However, the latter mutation perturbs quinone reduction leading to a diminished activity. D199^MT-ND1^ is part of a cluster of charged amino acid residues that are suggested to be important for efficient coupling of quinone reduction and proton translocation. A mechanism considering the role of D199^MT-ND1^ for energy conservation in complex I is discussed.

## Introduction

Mitochondria are essential organelles best known as powerhouses of the cell. The universal energy currency adenosine triphosphate (ATP) is produced in mitochondria via oxidative phosphorylation (OXPHOS), a process that couples electron transfer with ATP synthesis at the inner mitochondrial membrane. The electron transfer activities of complexes I to IV establish a protonmotive force (pmf) across the membrane that drives ATP synthesis by the ATP synthase. Complexes I (NADH:ubiquinone oxidoreductase) and II (succinate:ubiquinone oxidoreductase) reduce ubiquinone (Q) by oxidizing NADH and succinate, respectively. The reduced ubiquinol (QH_2_) is oxidized by complex III (cytochrome *bc*_*1*_) that in turn transfers the electrons to cytochrome *c* in the intermembrane space. Complex IV (cytochrome *c* oxidase), finally, accepts electrons from cytochrome *c* to reduce oxygen to water. The energy released during the electron transfer reactions is conserved by pumping protons from the mitochondrial matrix into the intermembrane space, thus generating the pmf^1–5^.

Complex I couples NADH oxidation and Q reduction with the translocation of protons across the membrane. It has a two-part structure with a peripheral arm catalyzing the electron transfer reaction and a membrane arm conducting proton translocation^6–10^. Mammalian complex I is made up of 45 subunits with 14 of them representing the core subunits required for catalytic activity. These 14 subunits are conserved between all species containing an energy-converting NADH:ubiquinone oxidoreductase and includes the seven subunits encoded by mitochondrial DNA (mtDNA) in humans.

OXPHOS defects are the most common inborn errors of metabolism most frequently associated with a dysfunction of complex I^11^. The deficiencies are genetically and clinically highly diverse including manifestations in early-onset neurodegenerative disorders such as mitochondrial encephalomyopathy and Leigh syndrome as well as lactic acidosis and cardiomyopathy^12^. As seven subunits of human complex I are encoded by mtDNA, mutations with an impact on structure and function of complex I may occur in both, the mitochondrial and the nuclear genome^3,13–16^. Complex I deficiencies caused by mutations in mtDNA are further influenced by their levels of heteroplasmy (i.e. the proportion of mutated *vs* wild type mtDNA) that show strong variations between patients cells and tissues^17–20^.

Modern next-generation sequencing techniques allow to readily obtain sequence data from patients suffering from mitochondrial diseases and lead to a steadily increasing number of identified pathological mutations^21^. However, biochemical data are mandatory to determine the impact of single mutations on the protein. These studies are complicated by the low amount of material available obtained by biopsy and from human cell lines^19,22,23^. Instead, well-characterized model systems such as *Escherichia coli*, *Paracoccus denitrificans*, and *Yarrowia lipolytica* have been established to identify effects of specific mutations on stability, assembly and electron transfer activity of complex I^24–29^. Although eukaryotic systems are evolutionary closer to the human enzyme, bacterial systems have the inestimable advantage that mutations found in mtDNA can be readily introduced into the bacterial genome. Protocols for mtDNA manipulation of the eukaryotic model organism *Saccharomyces cerevisiae* are available, but this organism does not possess respiratory complex I. As the architecture of the core subunits of complex I is conserved from bacteria to mammals, the structural minimal form of the *E. coli* enzyme was here used as model system. It has to be taken into account that *E. coli* lacks the so-called supernumerary subunits that stabilize mitochondrial complex I. This might influence the phenotype of a distinct mutation in the enzyme’s core structure. However, none of the positions studied here interact directly with a supernumerary subunit excluding such a specific effect. *E. coli* complex I is made up of 13 different subunits encoded by the *nuo*-genes that add up to a molecular mass of approximately 550 kDa^30,31^. In our experimental setup, the *nuo*-genes are expressed from a plasmid in a *nuo* null strain facilitating their mutation. An efficient and straight forward purification strategy was established allowing thorough analysis of the variant enzymes regarding their assembly, stability and catalytic activity^32–36^.

Here, we investigated the effect of two mutations that were identified in mtDNA of patients in the hospital center at the *Centre Hospitalo-Universitaire de Bordeaux* (Supplementary Text). A baby harboring the mutation m.13094T>C suffered from Leigh syndrome^37,38^. The mutation leads to a replacement of a conserved valine residue by an alanine residue (V253A^MT-ND5^) on subunit ND5. The second patient, an adult person suffering from nephropathy, deafness and diabetes mellitus carried an inversion of 7 bps (m.3902-3908inv7) in ND1. This inversion leads to the triple mutation D199G/L200K/A201V^MT-ND1^^39–41^, of which only D199^MT-ND1^ is conserved. We separately introduced the mutations at the conserved positions into a plasmid carrying the *E. coli* complex I genes and characterized the resulting mutant strains and the purified variants. The V253A^MT-ND5^ mutation strongly disturbed the assembly leading to the production of an inactive and instable complex. The D199G^MT-ND1^ mutation resulted in the production of a stable variant that showed a decreased electron transfer activity coupled to proton translocation. Remarkably, the variant exhibited a pronounced activation kinetics indicating perturbed quinone chemistry. Interestingly enough, D199^MT-ND1^ that is located between the two arms of the complex, is part of a cluster of charged residues that connects the quinone headgroup with the so-called E-channel that is discussed to play an essential role in proton translocation^42^.

## Results

### Conservation of amino acid residues

Conservation of positions D199^MT-ND1^ and V253^MT-ND5^ that were found mutated in the patients was confirmed by multiple sequence alignment using more than 50 sequences of prokaryotic and eukaryotic species (a selection is shown exemplarily in Supplementary Fig. 1). The alignment shows that position D199^MT-ND1^ is homologous to D213^H^ in *E. coli* and V253^MT-ND5^ to V259^L^ (the superscript refers to the name of the subunit of *E. coli* complex I). Hereafter, the *E. coli* nomenclature is used for clarity.

**Figure 1:**
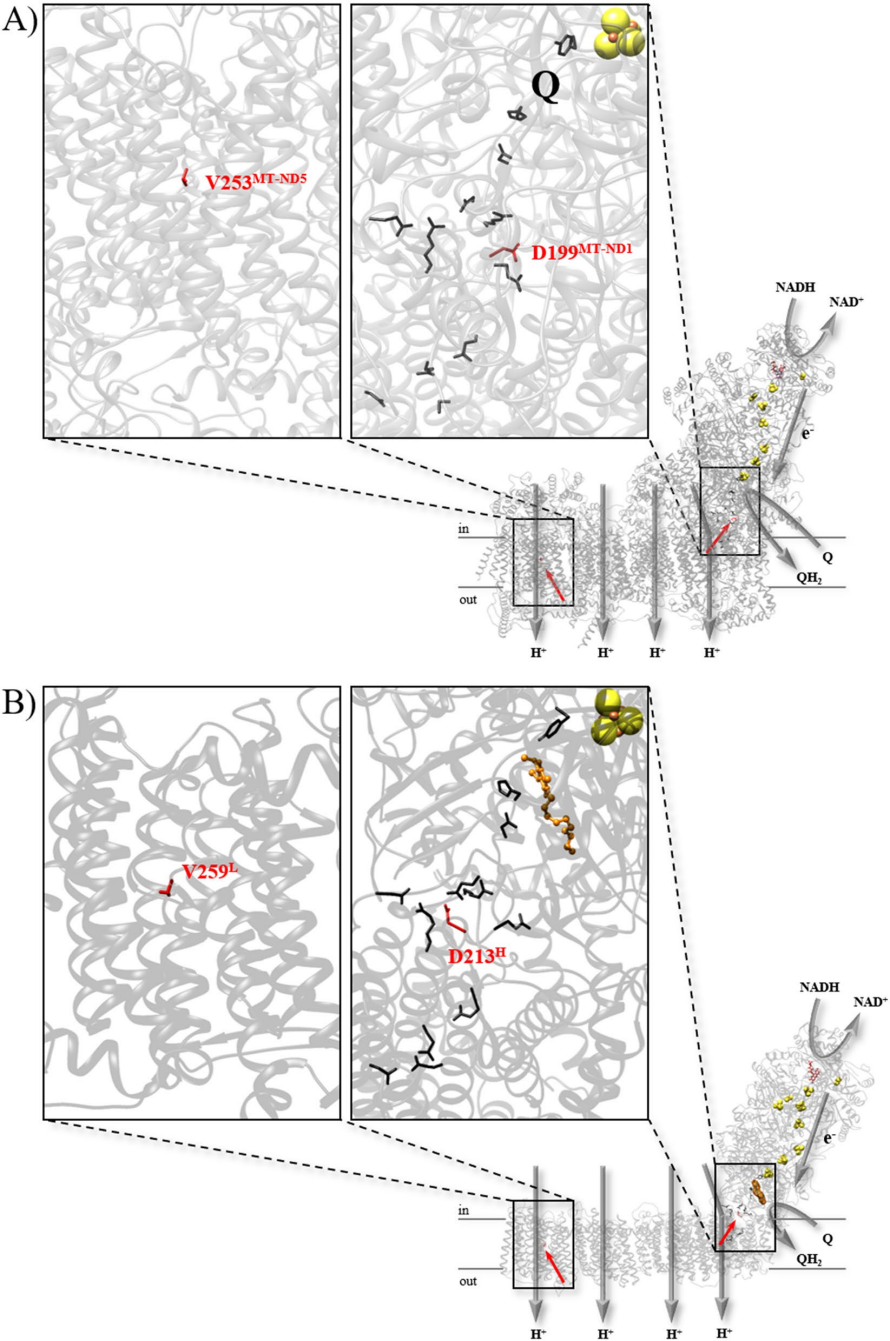
Structure of complex I from human (**A**) and from *T. thermophilus* (**B**) (PDB: 5XTD^81^ and 6I0D^82^)**.** For simplicity, subunits and residues of the bacterial complex are named according to the *E. coli* nomenclature. The positions of the mutations are shown in red; a close-up of the positions environment is shown in the insets. Residues of the area of charged amino acids around D199^MT-ND1^/D213^H^ and of the E-channel are shown in the right inset. Putative proton pathways are indicated with gray arrows, as well as NADH and quinone binding sites.

NADH is oxidized by FMN at the top of the peripheral arm and the electrons are transferred towards the membrane by a series of seven iron-sulfur clusters (Fig. 1). The distal cluster reduces Q in a unique cavity composed of subunits of both arms including NuoH. Together with NuoA, J and K, NuoH builds the so-called E-channel, a putative proton channel that is connected to the quinone binding site by a cluster of charged and polar amino acid residues^42,43^. D213^H^ is part of this cluster (Fig. 1). The chemistry of quinone reduction triggers proton translocation in the membrane arm through a series of four putative proton channels by a so far poorly understood mechanism^44^. NuoL is the most distal subunit of the membrane arm and harbors one of the proposed proton channels (Fig. 1). V259^L^ is located in TM helix 8 in close proximity to but not within the proposed proton channel^43^.

### Generation of mutants

The point mutations V259A^L^ and D213G^H^ were individually introduced into the pBAD*nuo*_His_ expression plasmid encoding the entire *E. coli nuo*-operon under the control of the inducible P_BAD_ arabinose promoter. Potential recombination with the chromosomal wild-type allele during cloning was excluded using strain DH5α∆*nuo* as cloning host^45^. The expression strain BW25113∆*ndh nuo*:*nptII*_FRT was transformed with expression plasmids either encoding the parental genes or those with the mutation. The expression strain chromosomally lacks the gene of the alternative NADH dehydrogenase (*ndh*)^46^ and the *nuo*-operon encoding complex I is replaced with the resistance cartridge (*nptII*) by λ-Red mediated recombination^35^. As consequence, NADH-induced activities of membranes reflect solely those of complex I encoded by the plasmid.

### Growth curves

**Figure 2:**
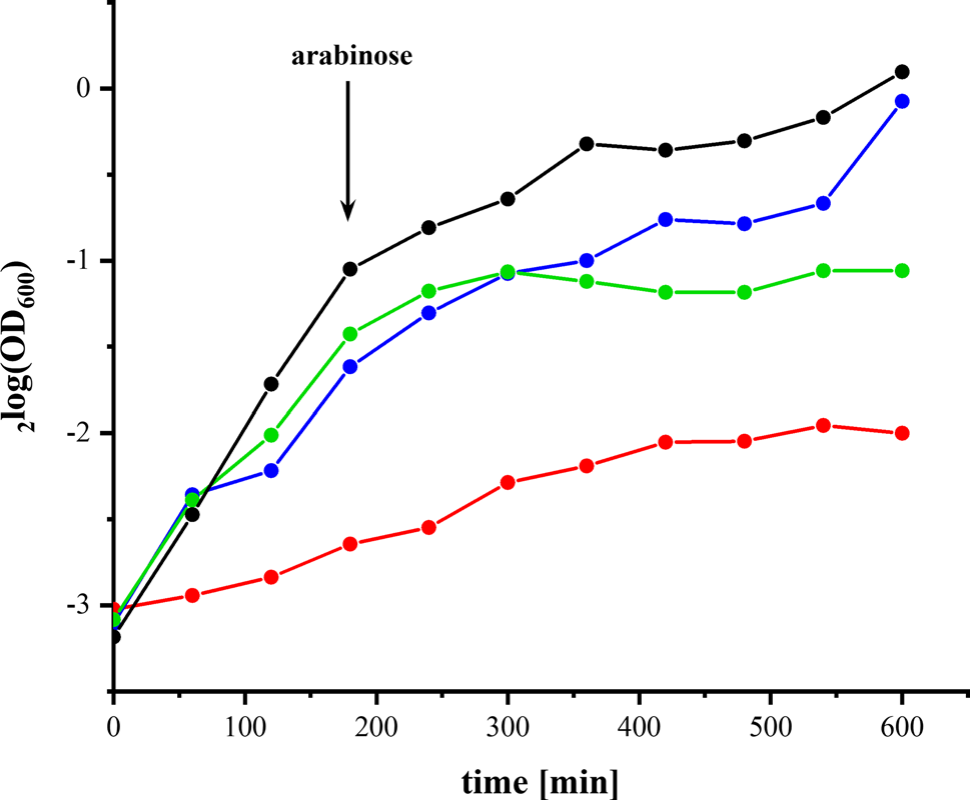
Growth of deletion strain BW25113*∆ndh nuo:nptII_FRT* (red) and strains BW25113*∆ndh nuo:nptII_FRT*/pBAD*nuo*_*His*_ (black), BW25113*∆ndh nuo:nptII_FRT*/pBAD*nuo *_*His*_*nuoF D213G*^*H*^ (blue) and BW25113*∆ndh nuo:nptII_FRT*/pBAD*nuo *_*His*_*nuoF V259A*^*L*^ (green) in minimal medium with acetate as sole carbon source. The black arrow indicates the induction of gene expression after 3 h growth by an addition of 0.02% arabinose.

The strains were grown in minimal medium using acetate as non-fermentable carbon source. Under this condition, fast growth of *E. coli* to high OD values depends on the presence of a functional complex I to maintain its NADH/NAD^+^ ratio low which is essential for preserving the TCA cycle^35^. As expected, the strain producing parental complex I grew the fastest, while the strain lacking both membranous NADH dehydrogenases showed the slowest growth rate (Fig. 2). Strains containing the plasmid grew faster than the double deletion strain even before induction due to a leaky expression (Fig. 2). The mutant strain producing the D213G^H^ variant grew as fast as the strain producing parental complex I, while the mutant strain containing the V259A^L^ variant entered the stationary growth phase shortly after induction of the extra-chromosomal *nuo*-genes. Thus, the mutation on NuoL seems to significantly affect NADH oxidation by complex I, while this is not the case for the mutation on NuoH.

### Activity of complex I and its variants in the cytoplasmic membrane

**Figure 3:**
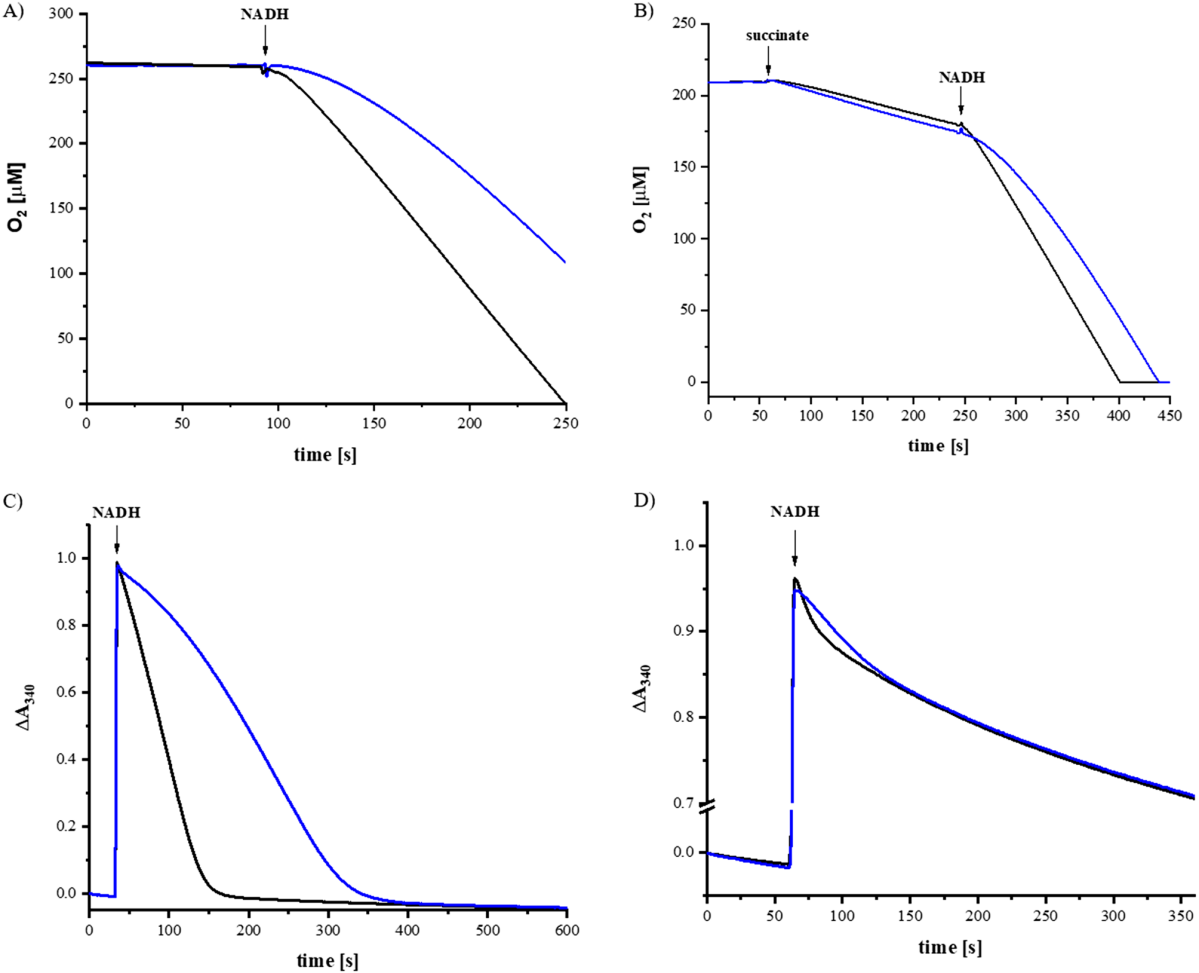
Activity of complex I (black) and the D213G^H^ variant (blue). (**A**) NADH oxidase activity of the membranes, (**B**) NADH oxidase activity after partial reduction of the Q-pool by an addition of succinate (0.034 ± 0.001 U/mg protein). The maximum NADH oxidase activity of membranes from the wild type was 0.413 ± 0.018 U/mg protein and that of membranes from the D213G^H^ mutant strain 0.194 ± 0.033 U/mg protein, (**C**,**D**) NADH:decyl-Q oxidoreductase activity of the preparations in the presence (**C**) and in the absence (**D**) of *bo*_*3*_ oxidase regenerating the quinol.

Cells were disrupted and cytoplasmic membranes were collected by differential centrifugation^47^. The amount of complex I in the membrane was estimated from the artificial NADH/ferricyanide oxidoreductase activity solely mediated by subunit NuoF containing the flavin cofactor^47^. This reaction is not coupled with Q reduction and proton pumping. The D213G^H^ mutant strain showed similar amounts of the variant protein as the parental strain producing complex I. The activity of the V259A^L^ mutant strain was halved indicating a decreased share of the variant protein in the membrane (Table 1). The physiological activity of the complex and the variants was determined by measuring oxygen consumption upon an addition of NADH (Fig. 3A). Here, the QH_2_ produced by complex I in a rate-limiting step is used to reduce oxygen to water by the quinol oxidases. Membranes from the V259A^L^ mutant strain showed no activity (Table 1). Thus, the NuoL mutant contained half of the amount of complex I compared to the parental strain but this variant was unable to catalyze the reduction of Q.

**Table 1:** NADH oxidase and NADH/ferricyanide oxidoreductase activity of cytoplasmic membranes from *E. coli* wild type and mutant strains.

	NADH oxidase activity		NADH/ferricyanide oxidoreductase activity	
	[U/mg]	[%]	[U/mg]	[%]
WT	0.421 ± 0.010	100 ± 2	2.00 ± 0.04	100 ± 2
V259A^L^	0.013 ± 0.002	3 ± 17	0.97 ± 0.09	50 ± 9
D213G^H^	0.302 ± 0.020	72 ± 7	1.88 ± 0.34	96 ± 18

Data were derived from three biological replicas; each replica was measured in triplicates. The standard deviation of the measurement is shown.

Membranes from the D213G^H^ mutant strain contained a functional complex I, but the apparent activity was decreased by a quarter (Fig. 3A, Table 1). Noteworthy, while the NADH oxidase activity of wild-type membranes started immediately after an addition of NADH, measurements with membranes from the D213G^H^ mutant strain showed a prominent lag-phase and the maximal turnover of 72% (compared to parental complex) was reached after approximately 1 min (Table 1; Fig. 3A). To examine whether the dissociation of the product QH_2_ from the complex was hampered in the mutant, the quinone pool was partially reduced by complex II due to an addition of succinate before complex I activity was initiated by an addition of NADH. A more reduced Q pool is expected to prolong the duration of the lag-phase. However, the prior addition of succinate had no significant effect on the duration and the course of the NADH-induced lag-phase indicating that product release is not disturbed in the mutant (Fig. 3B and see below).

### Assembly and stability of the variants

**Figure 4:**
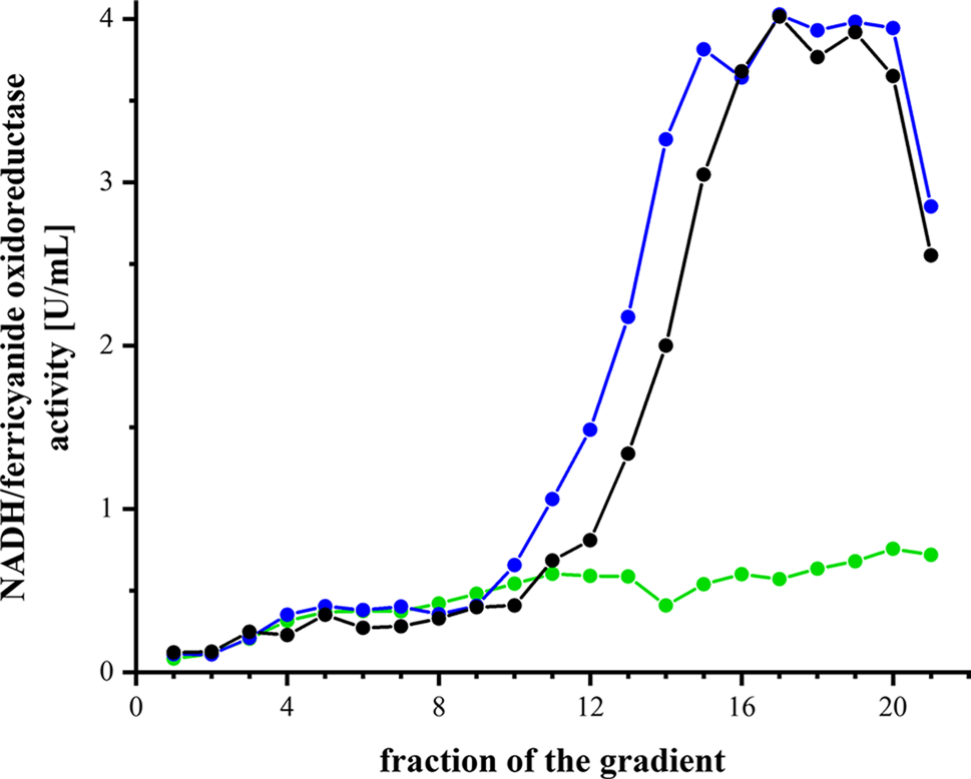
Sucrose gradient of detergent solubilized membranes from wild type (black) and the D213G^H^ (blue) and V259A^L^ (green) mutant strains. The NADH/ferricyanide oxidoreductase activity of each fraction is shown; the activities are normalized to 20 mg membrane protein extract applied on each gradient.

The stability of the complex and the variants in detergent was investigated by sucrose gradient density centrifugation (Fig. 4). Cytoplasmic membranes of cells grown in rich medium were incubated with DDM and the cleared extract was loaded onto a sucrose gradient. Solubilized membrane proteins were separated by centrifugation and the gradients were fractioned in 1 mL portions. NADH/ferricyanide oxidoreductase activity of each fraction was determined showing that wild type complex sedimented around fraction 16 as described earlier^47^. The activity profile of the gradient loaded with the extract from the D213G^H^ mutant strain was very similar: the peak was insignificantly broader and its position in the gradient within the experimental error. In contrast, no activity peak was detected in the gradient loaded with the extract from the V259A^L^ mutant strain (Fig. 4). To test whether a milder extraction could lead to an enrichment of the variant or an assembly intermediate, mutant membranes were treated with various low detergent concentrations and the NADH/ferricyanide oxidoreductase activity of the supernatant after high speed centrifugation was determined. It turned out that a substantial share of the activity was extracted with 0.2% DDM after 30 min (Supplementary Fig. 2). Accordingly, membrane extracts obtained after incubation with 0.2 and 0.8% DDM were loaded on a sucrose gradient as described above (Supplementary Fig. 2). However, at these mild conditions no activity peak from wild type complex I was detectable indicating that the DDM concentration was too low to extract the complex from the membrane. Accordingly, no activity peak was observed in the gradients loaded with the 0.2 and 0.8% extract from the mutant membranes (Supplementary Fig. 2). The activity in the detergent extract most likely derived from the detached peripheral arm of the complex. The peripheral arm is not stable on its own and decays^47^. Thus, the mutation V259A^L^ leads to an unstable complex that disintegrates upon detergent treatment. These findings are in line with the diminished NADH/ferricyanide oxidoreductase activity and the lack of NADH oxidase activity in this strain (Table 1).

### Isolation of complex I and of the D213G^H^ variant

**Figure 5:**
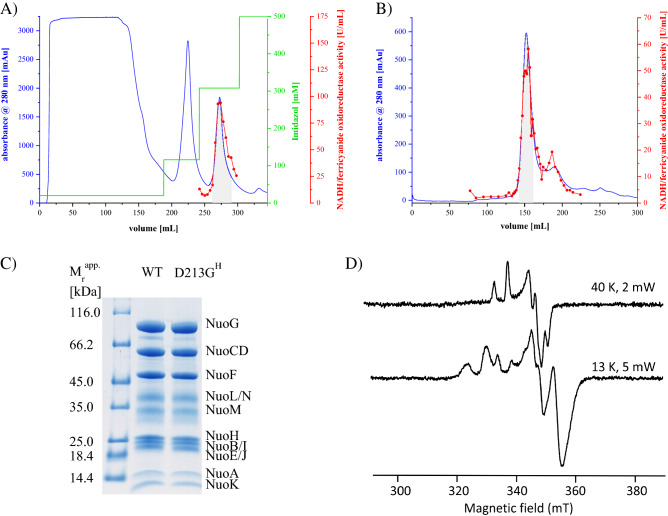
Preparation of complex I and the D213G^H^ variant. Elution profile of (**A**) the affinity-chromatography on ProBond Ni^2+^-IDA and (**B**) the size exclusion chromatography on Superose 6. Fractions indicated with a grey background were used in the next step. Preparations of the wild type and the variant showed virtually identical elution profiles. (**C**) SDS-PAGE of both preparations revealing the presence of all complex I subunits. The faint band at around 80 kDa is a proteolytic digestion product of NuoG. (**D**) EPR spectra of the preparation of the D213G^H^ variant reduced with NADH at 40 K and 2 mW (top), 13 K and 5 mW (bottom).

The complex was isolated from the parental strain and the D213G^H^ mutant strain grown in rich medium. Membrane proteins were extracted with the detergent LMNG and the complex was prepared to homogeneity by affinity- and size exclusion-chromatography (Fig. 5). In average, 7–9 mg protein was obtained from 50 g cells. Both preparations contained all complex I subunits as judged from SDS-PAGE and the protein band pattern of both preparations was virtually identical. The EPR spectra of the variant showed no differences to that of the complex (Fig. 5). Concentrated samples of both preparations were abruptly diluted in buffer to determine their stability by mass photometry^48,49^ (Supplementary Fig. 3). The molecular mass of the main peak in both samples was 733 kDa corresponding to complex I with bound detergent. This peak contained 77% of all binding events in both samples demonstrating that both preparations share the same stability. A decreased stability of the variant would result in a decreased number of binding events at that molecular mass.

### Catalytic activities of the preparations

The preparations of complex I and the D213G^H^ variant had similar specific NADH/ferricyanide oxidoreductase activities of 100 ± 10 U/mg (Table 2). The D213G^H^ variant showed about half of the NADH:decyl-Q oxidoreductase activity of the parental complex with *bo*_3_ oxidase as a quinone regenerating system (Table 2). Again, the kinetic trace of the variant exhibited a slow initial velocity that accelerated over time as observed in the measurement with membranes (Fig. 3C). In the absence of the Q regenerating system, QH_2_ is steadily enriched in the membrane. If the kinetic lag-phase of the variant is due to a disturbed release of the product, the lag-phase should be more pronounced in the absence of *bo*_3_ quinol oxidase. Under this condition, both preparations showed a much lower activity, however, the variant’s kinetic lag-phase remained unchanged (Fig. 3D). Thus, the mutation did neither influence NADH oxidation, nor the release of the product QH_2_. EPR spectra of the Fe/S clusters remained unchanged indicating an intact electron transfer in the peripheral arm. This indicates that Q reduction, which requires proton uptake, is most likely affected by the mutation.

**Table 2:** NADH:decyl-Q and NADH/ferricyanide oxidoreductase activity of complex I and the D213G^H^ variant in detergent.

	NADH:Q oxidoreductase activity		NADH/ferricyanide oxidoreductase activity	
	[U/mg]	[%]	[U/mg]	[%]
WT	41.0 ± 1.1	100 ± 3	94.0 ± 6.7	100 ± 7
D213G^H^	19.0 ± 1.4	46 ± 8	91.5 ± 5.6	97 ± 6

Data were derived from three biological replicas; each replica was measured in triplicates. The standard deviation of the measurement is shown.

**Figure 6:**
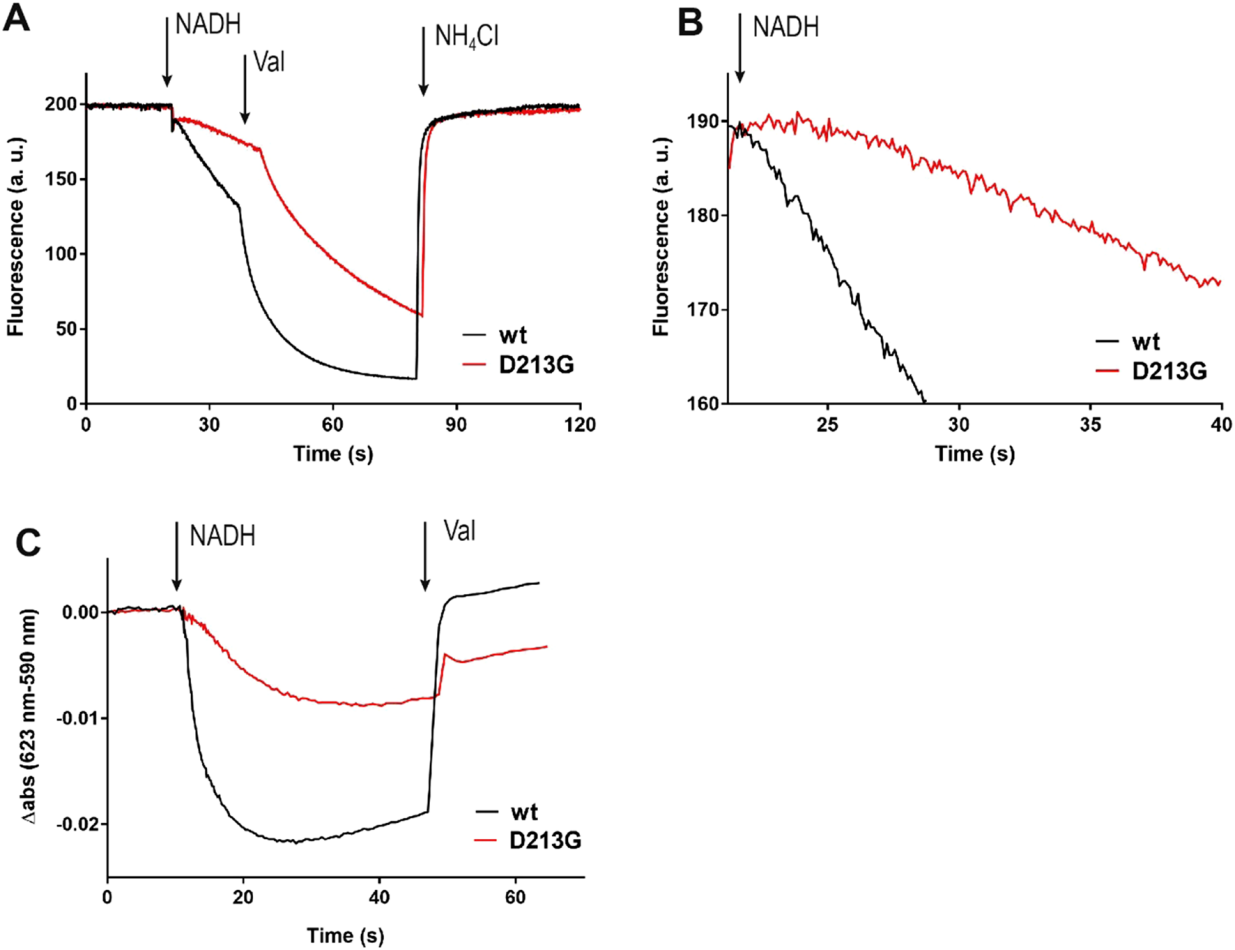
Generation of a pmf by *E. coli* complex I (black) and the D213G^H^ variant (red) reconstituted into liposomes. (**A**) Generation of a ΔpH measured as quench of the ACMA fluorescence. The reaction was started by addition of 130 µM NADH. Addition of valinomycin dissipates the Δψ and accelerates NADH turnover and proton pumping. Addition of 20 mM NH_4_Cl dissipated the proton gradient. (**B**) Close-up of A to reveal the lag in proton pumping activity in the D213G^H^ mutant. (**C**) Generation of a ΔΨ measured as the decrease of the oxonol VI absorbance difference (590–623 nm). The membrane potential was dissipated by addition of 200 nM valinomycin.

In order to measure NADH-driven proton translocation across membranes, the preparations of complex I and the D213G^H^ variant were reconstituted into preformed liposomes according to protocols established for *bo*_3_ oxidase and ATP synthase^76^. The orientation of the complex in proteoliposomes was estimated by measuring the NADH/ferricyanide oxidoreductase activity before and after an addition of 2% DDM. Both preparations showed a similar orientation with about 60% of the NADH binding site on the outside. Only these proteins are addressed in measurements as the substrate NADH is membrane impermeable. Proteoliposomes were incubated with decyl-Q for two minutes to allow its equilibration with the membrane. Proton pumping was initiated by the addition of 130 µM NADH that leads to an acidification of the liposome lumen and a membrane potential ∆Ψ that is inside positive. First, acidification of the vesicles was followed using the 9-amino-6-chloro-2-methoxyacridine (ACMA) fluorescence quenching measurements (Fig. 6A). Upon addition of NADH, fluorescence is quenched in both preparations however, the rate obtained with proteoliposomes containing the variant was diminished to about 50–70% depending on proteoliposome preparation. Importantly, the signals obtained with both preparations were sensitive to an addition of valinomycin, indicating that a counteracting membrane potential was built up during proton pumping. In addition, the delay in reaching maximal activity with the D213G^H^ variant was also observed in this type of measurement (Fig. 6B).

Generation of a membrane potential ∆Ψ (inside positive) was followed photometrically with the potential-sensitive dye oxonol VI (Fig. 6C). The signal observed with proteoliposomes with the D213G^H^ variant showed approximately one third of the amplitude of that with complex I. Both signals were fully sensitive to addition of the ionophore valinomycin that abolishes ∆Ψ in the presence of potassium. These experiments show that the D213G^H^ variant is capable of catalyzing redox-driven electrogenic proton-translocation. The observed rates of proton translocation and generation of ∆Ψ are in accord with the decreased NADH:decyl-Q oxidoreductase activities measured with the solubilized enzymes, suggesting that the NADH/H^+^ stoichiometry is not affected. The strong sensitivity of proton pumping towards valinomycin supports the idea of an intact coupling mechanism. However, the observed delay in all measurements points towards hampered quinone reduction.

## Discussion

The complex I mutations described here were previously identified as clinically relevant^37–41^. The m.13094T>C mutation exhibits variable neurological manifestations and is associated with an early mortality^37,38^ as with the patient reported here. This may be explained by the drastic effect of the V259A^L^ mutation in *E. coli*, namely a faulty assembly leading to a fragile and inactive complex I. V259^L^ is located in TM helix 8 in close proximity to a π-helix running from position 253 to 258^43^ and close to the most distal putative proton channel. π-Helices are commonly used to enhance protein functionality^50^. It was proposed that the π-helix in NuoL is an essential element to structure its core^51^. The stabilization of the protein structure is most likely disturbed by the mutation resulting in the drastic effects. Accordingly, the patients suffer from an imbalanced NADH/NAD^+^ ratio in mitochondria that down regulates the Krebs cycle^52^. Furthermore, the first coupling site in mitochondria is lost resulting in a decreased energy supply. These findings are in accordance with severe patient outcomes in childhood. Valente et al.^53^ reported data of cybrids derived from a child carrying the m.13094T>C mutation to a highly variable mutant load in skeletal muscle, lymphocytes and fibroblasts. The activity of complex I strongly correlated with the degree of heteroplasmy resulting in a nearly complete loss of complex I activity in cybrids that carry high mutant loads. The amount of complex I was clearly diminished in 80% heteroplasmic mutant cybrid cells and an assembly intermediate containing the membrane arm of the complex was accumulated in the membranes^53^. Unfortunately, an assembly intermediate lacking NADH dehydrogenase activity cannot be detected by our assays.

It is remarkable that a single point mutation in NuoL leads to the disintegration of the entire complex. Recently, we have shown that a deletion of *nuoL* in *E. coli* results in the assembly of the entire complex just lacking NuoL^54^ while C-terminal truncations of NuoL were tolerated and led to the assembly of the entire complex^54^. Thus, it is most likely that the central π-helix is an essential element to structure NuoL^51^ and that the mutation induced NuoL misfolding or destabilization that is transmitted to the other subunits of the complex. It was also reported that single point mutations in NuoL (e.g. R431^L^)^55^ and in NuoN (e.g. K217)^56^ fully disturbed the assembly of complex I.

The inversion of 7 bps on mitochondrial DNA (m.3902-3908inv7; MT-ND1) leads to a triple mutation on ND1 while maintaining the reading frame. One patient with the inversion found in several tissues such as heart, muscle and liver, died in infancy^40^. The patient reported here has a heteroplasmy of 67% in muscle, 20% in buccal epithelial cells and absent in blood. Other patients carry this mutation solely in muscles^39^. A morphological and enzyme histochemical analysis of skeletal muscle biopsy from patient 2 is shown in Supplementary Fig. 4. These patients experienced symptoms such as exercise intolerance, myalgia, deafness and nephropathy in their 40 s^39^. Only position D199 (human numbering) is conserved. Various other residues such as Phe, Gln and Asn are found at position L200 (human numbering). In position A201 (human numbering) Thr, Gln and even Pro are present. As D199 resides in a region with a high degree of sequence conservation, a change of all three amino acid residues in question might disturb the local structure. This structural effect might obscure the functional effect of the single D199G^MT-ND1^ mutation that kept structural changes at a minimum. Furthermore, the triple mutation D199G/L200K/A201V^MT-ND1^changes the overall negative charge of the short stretch to a positive one. However, it cannot be excluded that there is an additional influence of the other mutations caused by the inversion.

The D213G^H^ mutation in *E. coli* led to the assembly of a stable variant capable to catalyze a diminished electron transfer coupled with proton translocation. Accordingly, the mutation D199G^MT-ND1^ might have a milder effect in humans as it only slightly affects energy supply and the mitochondrial NADH/NAD^+^ ratio.

Mutations of *E. coli* residue D213^H^ were reported earlier^27,28^. The exchange to either a glutamate or an asparagine residue had little or no effects on the assembly of the complex but diminished its NADH:decyl-Q oxidoreductase activity by 60 and 40%, respectively^28^. Interestingly, D213E^H^, but not D213N^H^ showed also a lag-phase within the first minute of the measurement. In contrast, the D213A^H^ variant showed a disturbed assembly and a loss of 90% of the decyl-Q oxidoreductase activity^27^. Nevertheless, oxidation of d-NADH, a substrate specific for complex I, led to the generation of a membrane potential although with smaller amplitude than in wild type.

**Figure 7:**
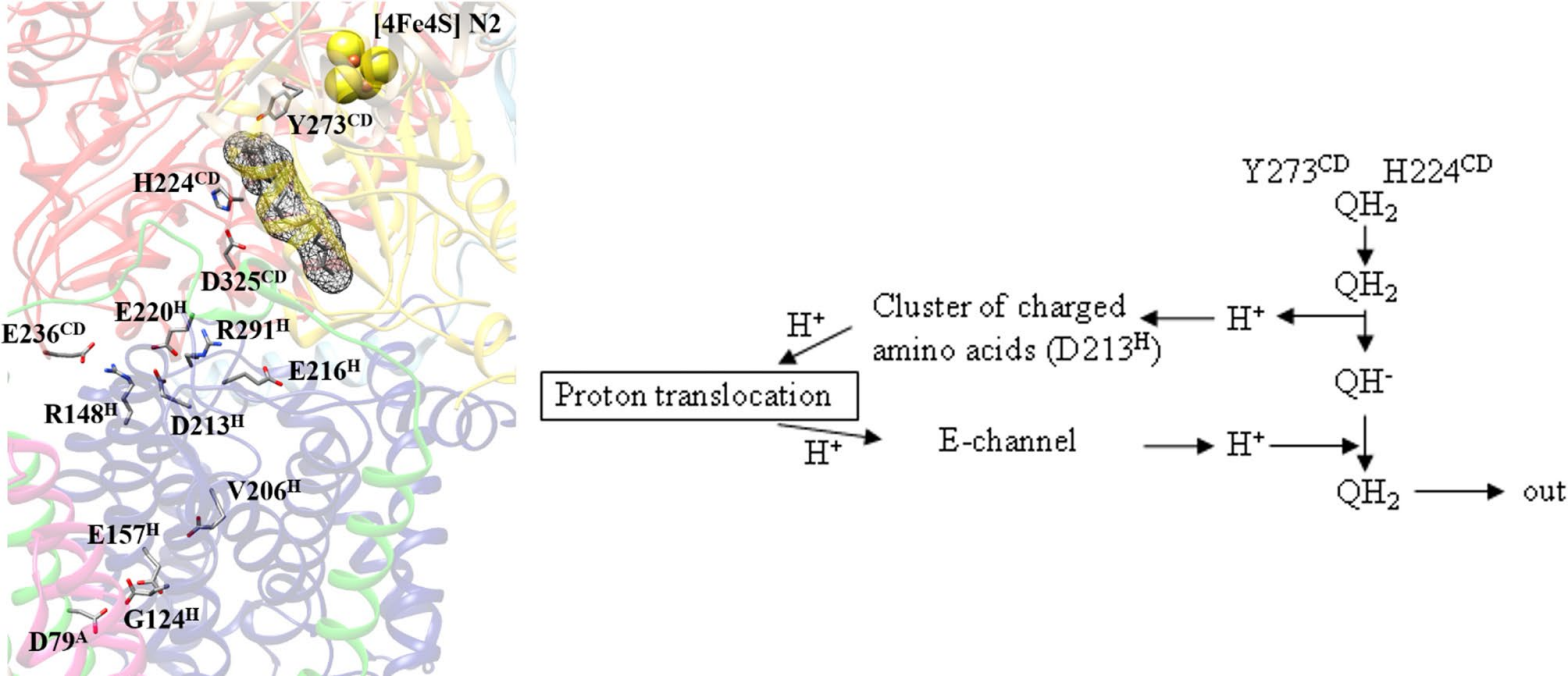
Proposed mechanism to activate proton pumping in respiratory complex I. The structure of the quinone binding site with the cluster of charged amino acids and residues of the E-channel are shown (*T. thermophilus* complex I, PDB: 6I0D^82^)**.** For clarity, residues are labeled according to the *E. coli* numbering. Q is bound close to iron-sulfur cluster N2 where it is reduced by N2 and protonated by Y273^CD^ and H224^CD^. According to the proposed model, the product QH_2_ moves to a second binding position in close vicinity to an area of charged amino acids, where it is deprotonated resulting in the formation of the QH^-^ anion, while the proton is transferred into the area of charged amino acids comprising D213^H^. This proton transfer activates proton translocation as described^44^ resulting in a surplus proton on an acidic amino acid of the E-channel after proton translocation. This proton is transferred to the QH^−^ anion leading to the formation of QH_2_ that is released from the enzyme.

We propose that the D213G^H^ and possibly also the D213E^H^ mutation influences quinone chemistry that drives proton translocation. According to computational and structural studies, the Q cavity provides at least two binding positions^5,43,44,51,57–62^. Q binds in 12 Å distance to the distal iron-sulfur cluster, N2, approximately 15 Å above the membrane surface^43,63^. The quinone headgroup is hydrogen-bonded by two conserved residues, namely Y273^CD^ and H224^CD^^64,65^. The human orthologues of NuoCD are two separated subunits called NDUFS3 and NDUFS2, respectively. It is reasonable to assume that Q is sequentially reduced by electrons from cluster N2 and protonated by these two residues. A recently discovered water-channel leading to this position may be used to re-protonate the tyrosine and histidine residues^51^. A hydrogen bond between H224^CD^ and D325^CD^ is broken upon deprotonation of H224^CD^, leading to a conformational move of D325^CD^ towards an area of charged residues, allowing QH_2_ to dissociate and move towards its second binding site^62,66^ (Fig. 7). QH_2_ movement may initiate proton pumping^42^ by a change of dipolar interactions within the membrane arm as described in detail by Kaila^44^. The area of charged amino acid residues including D213^H^ connects the quinone binding site with the E-channel, the proton translocation pathway closest to the peripheral arm^42,43^.

According to electrostatic calculations, the movement of QH_2_ towards its second binding site may alter the protonation state of nearby titratable residues^42,60,66–68^ possibly facilitating the deprotonation of QH_2_ to the QHˉ anion that is discussed to be a relevant intermediate. We propose that upon movement of D325^CD^, the cluster of charged amino acids acts as a putative proton relay system deprotonating QH_2_ (Fig. 7). The extracted proton is transferred to the E-channel initiating a cascade of changing dipolar interactions that continues all along the membrane arm. According to the theory, the ‘wave’ of changing dipolar interactions is reflected at the end of the membrane arm and travels back towards the peripheral arm, leading to proton uptake from the N-side and to proton release to the P-side. This process ends with a surplus proton that might be localized on an acidic amino acid residue of the E-channel. According to our model, the QHˉ anion formed at the cluster of charged amino acids moves to the second Q binding site where it is re-protonated and QH_2_ leaves the quinone binding cavity. From these considerations, it is obvious that the array of charged residues plays a crucial role both in the timing of proton coupled electron transfer and also in the efficient transfer of the anionic intermediate to the second binding site. Electron transfer and proton translocation activity of the D213^H^ variant showed a prominent lag-phase in which activity accelerated over time (Figs. 3 and 6). The localization of D213^H^ suggests that electron and proton transfer to Q and initial displacement of QH_2_ is unaffected also in the D213G^H^ variant. However, it can be envisioned that the mutation disturbs the finely tuned pK_A_ balance in the area of charged amino acids and either affects deprotonation of QH_2_ or proton release to the E-channel. MD-simulations and cryo-EM structures show that the area of charged residues is hydrated^51,68^, possibly enabling the insertion of a structural water at the mutated position G213^H^, representing a poor but sufficient substitute for the carboxylate group. Accordingly, the pronounced lag-phase of activity would be caused by a slow hydratization of this position. This hydratization is not possible with the more voluminous alanine variant reported in the literature^27,28^ leading to the pronounced inactivity. Hydratization is also not possible for the D213E^H^ and D213N^H^ mutants, but the hydrophilic nature of the side chains still allows for coupled reaction to occur.

A plethora of mtDNA mutations coding for complex I subunits are associated with the occurrence of mitochondrial disorders. Here, we applied a strategy to introduce clinically relevant mutations into bacterial complex I enabling the manipulation of genes that are mitochondrially encoded in eukaryotes. Other clinically relevant mutations with a mild patient outcome may lead to the identification of distinct changes that are helpful to clarify the mechanism of respiratory complex I.

## Methods

### Sequence alignments

Multiple Sequence Alignments were performed using ClustalX 2.1^69^. Default values were used for the penalties for opening and extending gaps.

### Strains, plasmids and oligonucleotides

A derivative of *E. coli* strain BW25113^70^ chromosomally lacking the gene *ndh* and in which the chromosomal *nuo* operon was replaced by a resistance cartridge (*nptII*)^35^ was used as host to overproduce complex I and its variants. *E. coli* strain DH5α∆*nuo*^45^ was used for site-directed mutagenesis. Oligonucleotides were obtained from Sigma-Aldrich (Supplementary Table 1). Restriction enzymes were obtained from Fermentas.

The plasmid pBAD*nuo*_*His*_^32^ was used to introduce the respective point mutations on *nuoL* and *nuoH* by site-directed mutagenesis according to the QuikChange protocol (Stratagene, La Jolla, CA, USA). Silent mutations were introduced generating a new restriction site near by the point mutation to identify positive clones by restriction analysis. Primer pairs nuoL_V259A and nuoH_D213G (Supplementary Table 1) were used to generate the plasmids pBAD*nuo *_*His*_*nuoF* D213G^H^ and *pBADnuo *_*His*_*nuoF* V259A^L^. The PCR was performed using the KOD Hot Start DNA Polymerase (Novagen). Mutations were confirmed by DNA sequencing (GATC Eurofins, Konstanz, Germany).

### Cell growth and isolation of cytoplasmic membranes

Strains were grown aerobically at 37 °C while agitating at 180 rpm. Minimal medium^71^ with 25 mM acetate as sole carbon source and baffled flasks were used. After 3 h growth, expression of the *nuo* operon was induced by an addition of 0.02% (w/v) l-arabinose. For protein preparation, cells were grown in auto-induction medium^72^ (1% (w/v) peptone, 0.5% (w/v) yeast extract, 0.4% glycerol, 25 mM Na_2_HPO_4_·2 H_2_O, 25 mM KH_2_PO_4_, 50 mM NH_4_Cl, 5 mM Na_2_SO_4_, 2 mM MgSO_4_·7H_2_O, 0.2% (w/v) l-arabinose, 0.05% (w/v) glucose, 30 mg/L Fe-NH_4_-citrate, 0.5 mM l-cysteine, 50 mg L^−1^ riboflavin) containing chloramphenicol (34 µg/ml) and harvested by centrifugation (5700×*g*, 15 min, 4 °C; Avanti J-26 XP, Beckman Coulter; Rotor JLA 8.1000) in the exponential phase yielding between 6 and 7 g cells/L. All further steps were carried out at 4 °C. The cell sediment was suspended in a fivefold volume buffer A (50 mM MES/NaOH, 50 mM NaCl, pH 6.0) containing 0.1 mM PMSF supplemented with a few grains of DNAseI and disrupted by passing three times through an EmulsiFlex-C5 (1000–1500 bar). Cell debris was removed by centrifugation (9500×*g*, 20 min, 4 °C; RC-5 Superspeed Refrigerated Centrifuge, Sorvall Instruments; Rotor A 8.24). Cytoplasmic membranes were obtained from the supernatant by ultra centrifugation (160,000×*g*, 60 min, 4 °C; LE-80K Ultrafuge, Beckman; Rotor 60 Ti). Sedimented membranes were suspended in an equal volume (1:1, w/v) buffer A^*^ (buffer A with 5 mM MgCl_2_) containing 0.1 mM PMSF.

### Electron transfer activity

Activity assays were performed at 30 °C. The NADH oxidase activity of cytoplasmic membranes was determined by a Clarke-type oxygen electrode (DW1, Hansatech) to monitor the decrease in oxygen concentration in the buffer. The electrode was calibrated by an addition of a few grains sodium dithionite to air saturated buffer. The difference before and after reduction was attributed to 237 mM oxygen^73^. The assay contained 5 µL cytoplasmic membranes (80–90 mg/mL) in 2 mL buffer A*. After equilibration, the reaction was started by an addition of 1.25 mM NADH. In order to partially reduce the Q-pool, the activity of complex II was initiated by adding 10 mM succinate. After three minutes complex I was activated by the addition of 1.25 mM NADH. The NADH/ferricyanide oxidoreductase activity was determined as decrease in the absorbance of ferricyanide at 410 nm with a diode-array spectrometer (QS cuvette, d = 1 cm, Hellma; TIDAS II, J&M Aalen) using an ε of 1 mM^−1^ cm^−1^^74^. The assay contained 10 µL membrane suspension or 0.1 µL complex I and 1 mM K_3_[Fe(CN)_6_] in buffer A*. The reaction was started by an addition of NADH (0.2 mM, final concentration). The NADH:decyl-Q oxidoreductase activity was measured as decrease of the NADH concentration at 340 nm using an ε of 6.3 mM^−1^ cm^−1^ (QS cuvette, d = 1 cm, Hellma; TIDAS II, J&M Aalen)^75^. The assay contained 60 µM decyl-Q, 2 µg complex I and a tenfold molar excess (5 µg) *E. coli* cytochrome *bo*_*3*_ oxidase^77^ (if appropriate) in buffer A*_MNG_ (buffer A* with 10% (v/v) glycerol, 0.005% (w/v) LMNG (2,2-didecylpropane-1,3-bis-β-d-maltopyranoside; Anatrace)). The reaction was started by an addition of 150 µM NADH.

### Sucrose gradient centrifugation

Membrane proteins were extracted by an addition of 1% (w/v) DDM (n-Dodecyl-β-d-Maltoside; Anatrace) to a membrane suspension (80–90 mg/mL) in buffer A*. After incubation for 1 h at 4 °C, the suspension was centrifuged for 20 min at 160,000×*g* and 4 °C (Rotor 60Ti, L8-M Ultrafuge, Beckman). 1 mL of the supernatant was loaded onto 24 mL gradients of 5–30% (w/v) sucrose in A*_DDM_ (buffer A* with 0.1% (w/v) DDM) and centrifuged for 16 h at 140,000×*g* (4 °C, Rotor SW28, L8-M Ultrafuge, Beckman). The gradients were fractionated into 1 mL aliquots and the NADH/ferricyanide oxidoreductase activities of each fraction was determined by following the decrease in ferricyanide absorbance at 410 nm with an Ultraspec spectrophotometer (Amersham Pharmacia Biotech, Munich, Germany)^47^. To determine the stability of the V259A^L^ variant, membranes (80 mg/mL) were incubated for 30 min at 4 °C with various DDM concentrations (0.1–1.0%; w/v), the suspension was centrifuged as described above and the NADH/ferricyanide activity of the supernatant was measured. According to the results, membrane proteins were extracted from the mutant membranes with 0.2 and 0.8% (w/v) DDM and extracted proteins were separated by sucrose gradient centrifugation as described above.

### Preparation of complex I and the variant

All steps were carried out at 4 °C as described^36^. LMNG is a final concentration of 2% (w/v) was added to a membrane suspension (~ 70 mg/mL) in buffer A*_pH6.8_. After incubation for 1 h at room temperature with gentle stirring, the suspension was centrifuged for 20 min at 160,000×*g* and 4 °C (Rotor 60Ti, L8-M Ultrafuge, Beckman). The supernatant was filtered (Filtropur S0.45; Sarstedt), diluted to 150 mL, adjusted to 20 mM imidazole and applied to a 35 mL ProBond Ni^2+^-IDA column (Invitrogen) equilibrated in binding buffer (A*_MNG_ with 20 mM imidazole and pH 6.8). The column was washed with binding buffer and subsequently with binding buffer containing 116 mM imidazole until the absorbance at 280 nm dropped below 500 mAu. Bound proteins were eluted with binding buffer containing 308 mM imidazole. Fractions containing NADH/ferricyanide oxidoreductase activity were pooled and concentrated by ultrafiltration in 100 kDa MWCO Amicon Ultra-15 centrifugal filter (Millipore). The concentrate was applied onto a Superose 6 size exclusion chromatography column (300 mL, GE Healthcare) equilibrated in buffer A^*^_MNG_. The fractions with highest NADH/ferricyanide oxidoreductase activity were pooled and concentrated as described above. The protein was either directly used or stored at − 80 °C.

### Preparation of liposomes

*E. coli'.* polar lipids (25 mg/mL in CHCl_3_; Avanti) were evaporated and dissolved in the fivefold volume lipid buffer (5 mM MES/NaOH, 50 mM NaCl, pH 6.7)^76^. The suspension was frozen in liquid nitrogen and thawed at 29 °C seven times to get unilamellar vesicles. The liposomes were extruded by at least 21 passes through an extruder (0.1 µM polycarbonate membrane, Mini Extruder; Avanti). For reconstitution, ~ 0.5 mg complex I was mixed with reconstitution buffer (1:3; v/v) (10 mM Bis–tris-propane/MES, 100 mM KCl, 73 mM sucrose, 2.5 mM MgSO_4_, 0.05% (w/v) l-α-phosphatidylcholine, 1.1% (w/v) *n*-octylglucoside, 0.6% (w/v) sodium deoxycholate, 0.6% (w/v) sodium cholate, pH 7.5) and incubated for 5 min on ice. 250 µL liposomes were mixed with 8 µL sodium cholate (20%, w/v) and the liposomes and complex I in reconstitution buffer were mixed and incubated for 20 min at room temperature with occasional flicking of the tube. Liposomes were formed using a size exclusion column (PD-10 Desalting Column, 8.3 mL, Sephadex G-25, GE Healthcare) equilibrated in lipid buffer to remove excess detergent. The eluate (1.2 ml) was centrifuged (4 °C, 200,000×*g*, 45 min) and sedimented proteoliposomes were gently re-suspended in 500 µL lipid buffer.

### Membrane potential and proton gradient

The generation of ∆pH was determined by monitoring the fluorescence quenching of the pH sensitive dye 9-amino-6-chloro-2-methoxyacridine (ACMA, Sigma). The assay contained 100 µM decyl-Q (Sigma), 0.2 µM ACMA and 50 µL proteoliposomes in ACMA buffer (10 mM Bis–tris-propane/MES, pH 6.75, 100 mM KCl and 2 mM MgCl_2_). The reaction was performed at 25 °C and started by addition of 200 µM NADH. The fluorescence was detected with a Cary Eclipse fluorescence spectrometer (Agilent), using excitation and emission wavelengths of 410 nm and 480 nm, respectively. An addition of 20 mM NH_4_Cl was used to dissipate the ∆pH.

The generation of *∆*Ψ was determined by monitoring the changes in absorption of the potential-sensitive dye oxonol VI (Sigma). The assay contained 1 µM oxonol VI, 50 µM decyl-Q and proteoliposomes in oxonol buffer (10 mM MES/KOH, pH 6.75, 2 mM MgSO_4_, 100 mM KCl, 10 mM NH_4_Cl)^76^. The reaction was performed at 25 °C and started by addition of 100 µM NADH. The absorbance at 590–623 nm was measured with a diode-array spectrometer (QS cuvette, d = 1 cm, Hellma; TIDAS II, J&M Aalen). An addition of 200 nM valinomcyin was used to dissipate *∆*Ψ.

### Other analytical procedures

Protein concentration was determined according to the biuret method using BSA as a standard^78^. The concentration of purified complex I was determined by the difference of absorbance at 280–310 nm (TIDAS II, J&M Aalen) using an ε of 781 mM^−1^ cm^−1^ as derived from the amino acid sequence^79^. SDS-PAGE (sodium dodecyl sulfate–polyacrylamide gel electrophoresis) was performed according to Schägger^80^ with a 10% separating gel and a 3.9% stacking gel. EPR spectra were recorded at 40 and 13 K with a Bruker EMX 6/1 spectrometer operating at X-band^33^. The samples were reduced by an addition of 1.000-fold molar excess NADH and a few grains of dithionite. Mass photometry was measured with a OneMP (Refeyn) as described^48,49^. One µL of a concentrated sample (1 µM) of the preparations of complex I and the D213G^H^ variant were diluted with A*_MNG_ buffer to 50 nM on the cover slip and after autofocus stabilization, movies of 60 s duration were recorded. Data were acquired with AcquireMP (Refeyn Ltd., v.1.2.1).

## Supplementary Information

Supplementary Information.

## Data Availability

The source data underlying Figs. 2, 3, 4, 5, 6 are provided as a Source Data file. Other data are available from the corresponding authors upon reasonable request.
