# Supplementary material for: Biochemical consequences of two clinically relevant ND-gene mutations in Escherichia coli respiratory complex I
Source: Sci Rep. 2021 Jun 16;11:12641. doi: 10.1038/s41598-021-91631-3 (PMC8209014; doi:10.1038/s41598-021-91631-3)
Supplement: Supplementary file 1 — Supplementary Information. [file 41598_2021_91631_MOESM1_ESM.docx]

Supplementary Information

**Biochemical consequences of two clinically relevant ND-gene mutations in *Escherichia coli* respiratory complex I**

Franziska Nuber, Johannes Schimpf, Jean-Paul di Rago, Déborah Tribouillard-Tanvier, Vincent Procaccio, Marie-Laure Martin-Negrier, Aurélien Trimouille, Olivier Biner, Christoph von Ballmoos and Thorsten Friedrich

Supplementary Text

**Case descriptions**

A baby who had been initially well-being until 7 months of age was referred for an alteration of the general state, a psychomotor regression and failure to thrive. These symptoms were associated with recurrent infectious episodes. Cerebro-medullary MRI showed a T2 hyper signal of the lenticular nuclei and to a lesser degree of the anterior part of the caudate nuclei, associated to a moderate peak of lactates evoking an underlying metabolic pathology. Muscle biopsy showed a subsarcolemmal accumulation of mitochondria, with an increase of the succinate dehydrogenase and cytochrome oxidase activities. Respiratory chain complex analysis showed a slight decrease of complex III and complex IV activities. Sequencing of the total mtDNA by NGS revealed the variant m.13094T>C in MT-ND5 gene (NC_012920.1: c.MT-ND5:758T>C, p.ND5:(Val253Ala) ) with low heteroplasmic rate (10%) in the muscle and blood. At the time of the molecular diagnosis, the status of this variant in the MITOMAP database was referred as “reported” and prompted us to study the functional consequences of this variant on complex I activity.

An adult patient was referred for a kidney pre-transplant assessment. This patient had a sensorineural hearing loss since childhood, mild intellectual disability, and insulin dependent diabetes mellitus, long-standing arterial hypertension and end-stage kidney disease. Histological renal biopsy was in favor of diabetic nephropathy and nephroangiosclerotic lesions. The patient additionally had a moderate axonal sensory polyneuropathy and myoclonia had recently developed. The muscle biopsy showed ragged red fibres and cytochrome oxidase negative muscle fibers which were in favor of mitochondrial disease. Sequencing of the total mtDNA by NGS was performed as described elsewhere (doi: 10.1111/cge.13670). It revealed the pathogenic inversion m.3902-3908inv in MT-ND1 gene (NC_012920.1: c.MT-ND1:596_602inv, p.ND1:(Asp199_Ala201delinsGlyLysVal)) with a variable heteroplasmic rate in the different tissues studied: 67% in muscle, 20% in epithelial cheek mucosa, undetectable in blood (Supplementary Fig. 4).

A)


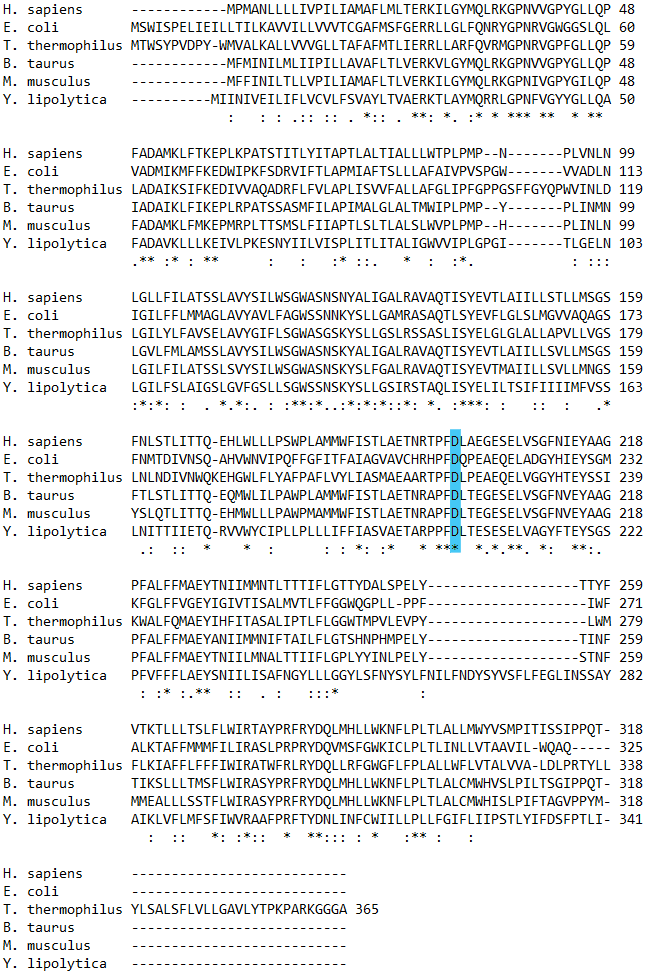


B)


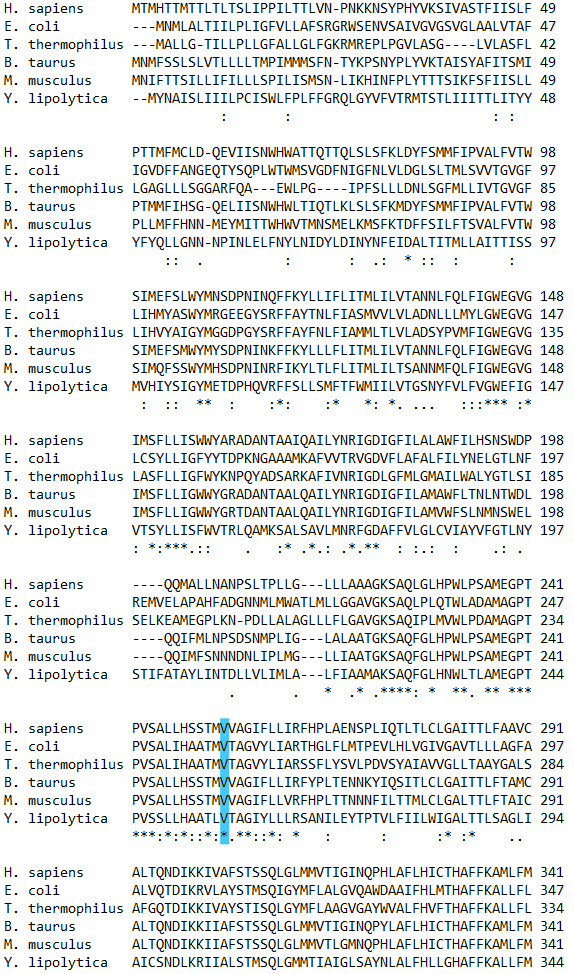


**Supplementary Figure 1 | Multiple sequence alignments of A) NuoH and B) NuoL.** The sequences from Homo sapiens, E. coli, T. thermophilus, Bos taurus, Mus musculus and Y. lipolytica are shown exemplarily. 85 and 58 sequences, respectively, were used to generate the alignments. The positions of residues D199 and V253 (human numbering) are highlighted in blue. Residues marked with a star are conserved through the aligned organisms.


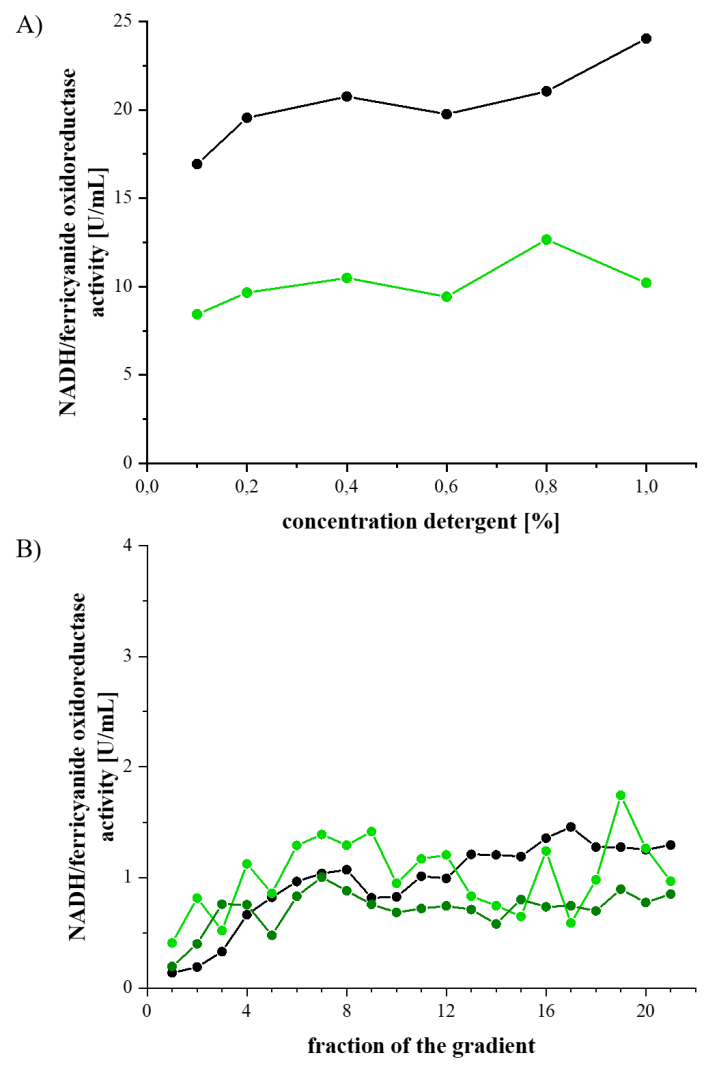


**Supplementary Figure 2 | Extraction of cytoplasmic membrane proteins with low detergent concentrations.** A) NADH/ferricyanide oxidoreductase activity of the supernatant after high speed centrifugation of the detergent extracts of membranes from wild type (black) and from the V259A^L^ mutant strain (green) incubated with various low DDM concentrations. The mean value of three measurements is shown. Similar curves were obtained after 60 minutes incubation. B) Sucrose gradient of detergent solubilized membranes from the wild type (0.8% DDM, 30 minutes, black) and from the V259A^L^ mutant strain (0.2% DDM, light green and 0.8% DDM, dark green, both after 30 minutes incubation). The NADH/ferricyanide oxidoreductase activity of each fraction is shown; the activities are normalized to 20 mg membrane protein extract applied on each gradient.


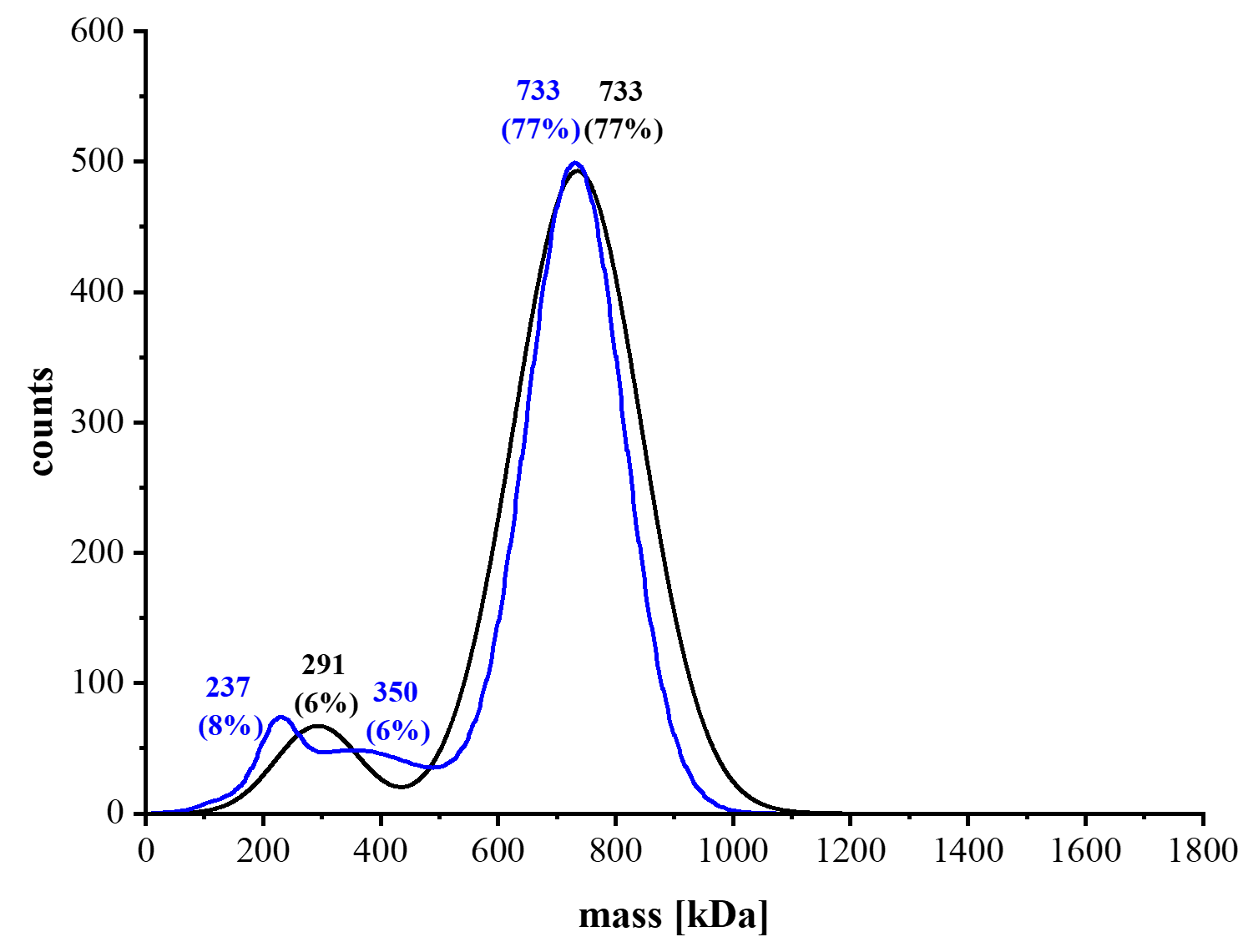


**Supplementary Figure 3 | Gaussian fitting to mass distribution plots of the preparation of complex I (black) and the D213G^H^ variant (blue) as obtained by mass photometry.** One µL (1 µM) protein were diluted in 20 µL A^*^_MNG_ and binding events with different masses were counted. For both preparations, 77% of all binding events appeared in the 733 kDa peak. The measurement of the D213G^H^ variant showed two additional peaks of low intensity (8 and 6%, respectively) at 237 and 350 kDa and that of complex I one additional peak at 291 kDa (6%).

A) B) C)


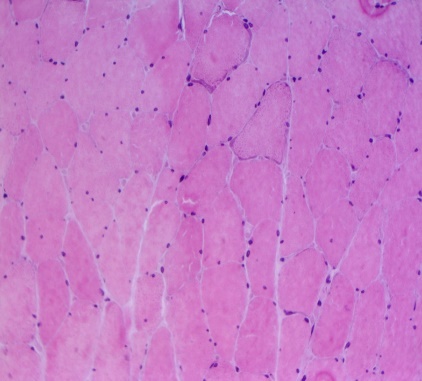

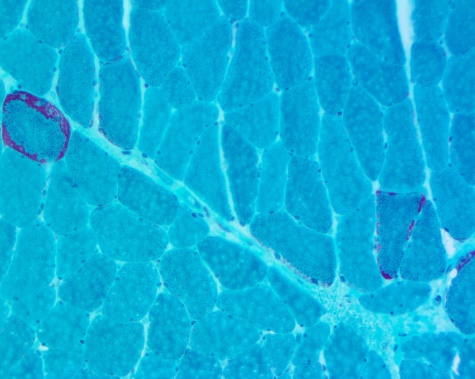

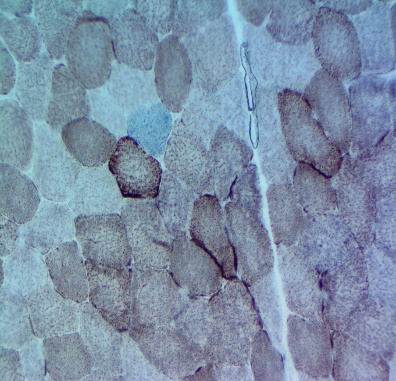


**Supplementary Figure 4 | Morphological and enzyme histochemical analyses of skeletal muscle biopsy in patient 2.** A) hematoxylin and eosin: Some fibers showed subsarcolemmal basophilic granulations. B) Modified Gomori trichrome stain: Few Ragged red fibers were observed. C) Cytochrome c oxidase (COX) and succinate dehydrogenase (SDH) double enzyme histochemistry: Rare COX deficient fibers were observed, more numerous fibers with a COX and SDH increase were present. Magnification 200x.

**Supplementary Table 1 | Oligonucleotides used for site-directed mutagenesis.** Newly generated codons are shown in bold, exchanged bases in italics. To proof the newly generated codons, silent mutations were inserted. The restriction sites are underlined. The ‘check’ oligonucleotides were used for DNA sequencing.

| **Oligonucleotides** | **sequence 5’-3’** | **Restriction site** |
| --- | --- | --- |
| nuoL_V259A_fwd | GATCCACGCCGCAACCATG**GCG**AC*T*GC*A*GGTGTC  TACCTGATCGCCCG | *PstI* |
| nuoL_V259A _rev | CGGGCGATCAGGTAGACACCTGCAGTCGCCATGG  TTGCGGCGTGGATC |  |
| nuoH_D213G_fwd | GTATGTCACCGTCACCCGTTT**GGC**CAGCCGGAAG  C*T*GAGCAGGAACTGGCGGATGGTTAC | *Bpu1102I* |
| nuoH_D213G _rev | GTAACCATCCGCCAGTTCCTGCTCAGCTTCCGGCT  GGCCAAACGGGTGACGGTGACATAC |  |
| Check_ nuoL_V259A | TGCGGTCGGTAAATC |  |
| Check_ nuoH_D213G | CAACATGACCGACATC |  |
